# Symmetry Is Related to Sexual Dimorphism in Faces: Data Across Culture and Species

**DOI:** 10.1371/journal.pone.0002106

**Published:** 2008-05-07

**Authors:** Anthony C. Little, Benedict C. Jones, Corri Waitt, Bernard P. Tiddeman, David R. Feinberg, David I. Perrett, Coren L. Apicella, Frank W. Marlowe

**Affiliations:** 1 School of Psychology, University of Stirling, Stirling, United Kingdom; 2 School of Psychology, University of Aberdeen, Aberdeen, United Kingdom; 3 Department of Zoology, University of Oxford, Oxford, United Kingdom; 4 School of Computer Science, University of St Andrews, St Andrews, United Kingdom; 5 Department of Psychology, McMaster University, Ontario, Canada; 6 School of Psychology, University of St Andrews, St Andrews, United Kingdom; 7 Department of Anthropology, Harvard University, Cambridge, Massachusetts, United States of America; 8 Department of Anthropology, Florida State University, Tallahassee, Florida, United States of America; University of Victoria, Canada

## Abstract

**Background:**

Many animals both display and assess multiple signals. Two prominently studied traits are symmetry and sexual dimorphism, which, for many animals, are proposed cues to heritable fitness benefits. These traits are associated with other potential benefits, such as fertility. In humans, the face has been extensively studied in terms of attractiveness. Faces have the potential to be advertisements of mate quality and both symmetry and sexual dimorphism have been linked to the attractiveness of human face shape.

**Methodology/Principal Findings:**

Here we show that measurements of symmetry and sexual dimorphism from faces are related in humans, both in Europeans and African hunter-gatherers, and in a non-human primate. Using human judges, symmetry measurements were also related to perceived sexual dimorphism. In all samples, symmetric males had more masculine facial proportions and symmetric females had more feminine facial proportions.

**Conclusions/Significance:**

Our findings support the claim that sexual dimorphism and symmetry in faces are signals advertising quality by providing evidence that there must be a biological mechanism linking the two traits during development. Such data also suggests that the signalling properties of faces are universal across human populations and are potentially phylogenetically old in primates.

## Introduction

Increasingly attention is being paid to the complexity of animal signalling [1]. Many animals display multiple traits and assess multiple signals. Multiple traits may be signals of the same factor, and so serve to enhance the accuracy with which receivers assess a single factor, or else signal different facets of an individual's quality [2]. In terms of sexual selection, signalling traits can be divided by their role in intrasexual (same-sex competition) and intersexual (choices of the opposite-sex) selection. While faces are likely to play a role in same-sex competition [3], it is the later form of sexual selection that has been most prominently applied to research on human facial attractiveness.

Darwin [4] laid out the first notions of how evolution of traits by preference could occur. Self-reinforcing, or “runaway”, selection [5] may explain certain traits. After a preference for any particular trait has arisen, for example, a preference for long tails in a bird species, females begin to reproduce with males in possession of long-tails to produce offspring with both genes for long tails (in males) and genes for a preference for long tails (in females). A feedback loop between genes for traits and preferences produce stronger preferences and ever more elaborate expression of traits. The initial preference could come from a sensory disposition evolved for another purpose [6] and hence arbitrary. The idea that male or female morphology may be attractive because it exploits an already existing preference in the opposite-sex has been called the perceptual or sensory bias view [7].

In contrast to such views, indicator mechanisms of sexual selection propose that certain traits are preferred because they are associated with either phenotypic or genotypic quality [8] and therefore act as cues and hence can be signals of quality. A key concept in indicator mechanisms is the notion of handicaps. Individuals may find mates who carry a costly handicap more attractive because the fact they have survived with the handicap is an indicator of their genetic quality [9]. Many traits also require energy to produce and so individuals must be in good condition to afford their production. Handicaps can then be ‘honest’–low quality individuals cannot ‘fake’ such traits. Individuals who choose partners in possession of such traits will produce more offspring than those who do not.

An important question is whether particular traits are driven by indicator mechanisms or are driven by arbitrary preferences. Researchers have suggested that different signals of the same quality should inter-correlate [10], [11], which would support indicator mechanisms in their evolution. For example, in humans, the judged attractiveness of female bodies correlates with facial attractiveness [11] and the pitch of female voices also positively predicts facial attractiveness [12]. Both studies suggest that the three traits measured are in part signalling one aspect of quality. Such a relationship should come about because the underlying quality advertised by one trait will also be reflected in other traits. If traits advertise discrete aspects of quality, then there is no apriori reason to expect such traits to co-vary. Theories suggesting that traits are being driven by perceptual bias or via arbitrary runaway selection also do not predict co-variation.

Two important traits thought to relate to mate-quality in many animals are symmetry and sexual dimorphism [13], [14]. Fluctuating asymmetry (FA) [15] is thought to reflect an individual's ability to maintain the stable development of their morphology under the prevailing environmental conditions. Fluctuating asymmetry is a useful measure as it subsumes a large amount of individual variation in development, reflecting differences in genetic (e.g., inbreeding, mutation, and homozygosity) and environmental (e.g., nutrient intake, parasite load) factors [16]. While the issue is controversial [17], many studies do show links between symmetry and quality including factors such as growth rate, fecundity, fertility and survivability [16], [18], [19] and one study has shown that symmetry in both men and women is negatively related to self-reported health problems [20]. Potentially, any link between symmetry and quality, no matter how weak, may be sufficient to create a selection pressure to choose symmetric mates. Symmetry in human faces has then been suggested to be a cue to heritable fitness benefits [21], [22] and studies of real [23], [24] and manipulated faces [22], [25] show that symmetry is found attractive. Facial symmetry is found attractive in different human cultures [26] and in monkey species [27].

In some species sexually dimorphic traits advertise genetic quality [14]. Larger jawbones, more prominent cheekbones, and thinner cheeks are all sexually dimorphic features in human faces characteristic of males [28], [29]. Such masculine features are associated with higher testosterone in males [30] while feminine features are associated with higher oestrogen in females [31]. Secondary sexual characteristics may be linked to parasite resistance because the sex hormones which influence their growth, particularly testosterone, lower immuno-competence [32]. Larger secondary sexual characteristics should be related to a healthier immune system because only healthy organisms can afford the high sex hormone handicap on the immune system that is necessary to produce them [33]. There is evidence in humans that testosterone acts as an immunosuppressant [34] but the data for women is less clear (see discussion). Testosterone may have a greater impact on immune function than oestrogen making sexually dimorphic features more costly for males.

Perceived masculinity in human faces is positively correlated with males' long-term health as assessed from medical records [35] and from self-reports [20]. Sexual dimorphism may also be linked to other mechanisms of quality advertising through links with testosterone, which influences behaviour [36]. In women femininity may also be linked to fertility through an association with oestrogen [31]. Sexual dimorphism in faces, another proposed marker of genetic quality [21], [29], [37], also influences preferences. Males prefer feminised female faces and females show increased preferences for masculinity in contexts consistent with masculinity signalling some aspect of quality [38], [39].

If symmetry and masculinity honestly indicate the quality of individuals, high quality individuals should develop large sexual ornaments which have little asymmetry. There is evidence for this within and across bird species where larger ornaments, such as tails, tend to be more symmetrical than smaller ornaments [13]. Associations between symmetry and trait size are more consistent with indicator models than an arbitrary process [8], [13]. If quality was unrelated to size and symmetry we would expect the cost of ornamentation to create developmental stress for their owners leading to increased asymmetry in large ornaments. However, if only high quality individuals are capable of bearing the handicap of growing large traits or symmetric traits we would expect size and symmetry of traits to correlate.

If symmetry and sexual dimorphism in faces indicate quality then a positive correlation between symmetry and sexual dimorphism would be predicted. Evidence for associations between symmetry and sexual dimorphism in men and women is equivocal, however [23], [24], [40], [41], and as of yet only city-based student samples have been examined.

Here we examined the relationship between measured facial symmetry and facial sexual dimorphism in human population samples from Europe and from an environment likely to reflect humans living under more evolutionary relevant conditions (the Hadza of Tanzania, Africa) as well as in a non-human primate (rhesus macaques, *Macaca mulatta*). We measured facial symmetry and sexual dimorphism from landmark points and tested for relationships between symmetry and sexually dimorphic proportions. We also tested if composites of symmetrical faces within each sample were perceived as being more sex-typical than composites of asymmetric faces.

## Materials and Methods

### Photographs

For the European images, male (177 individuals) and female (318 individuals) participants had their photograph taken in the laboratory with a digital camera under standardised lighting conditions. Participants were asked to pose with a neutral expression and to look directly into the camera to produce front on facial photographs. Participants were asked not to smile and to relax their face during photographs. Neutral expressions (as posed by our participants) can be seen in the average faces presented later. All individuals were less than 30 years old (ranging from 17–29, mean = 20.6, SD = 2.2). Participants were UK based university students who volunteered to take part in psychology studies and were primarily UK residents. The photographs were taken at the universities of Liverpool, Stirling, and St Andrews. Written consent was obtained for all participants and the collection of photographs was approved by relevant ethics committees at each institution.

The macaque and Hadza images could not be collected under laboratory conditions. For the macaque images, a digital video camera was used to capture images of adult males (105 individuals) and females (111 individuals) from the free-ranging population of rhesus macaques on Cayo Santiago, Puerto Rico. Only full-face images with neutral expressions were used, taken from video footage. All macaques had identifying tattoos, which were noted during image acquisition by CW, ensuring that all individuals included were unique. Images were collected from Cayo Santiago field station, the Primate Ecology Section of the National Institutes of Health Laboratory of Perinatal Physiology, which abides by US laws and practices in the ethical treatment of animals.

For the Hadza images, male (67 individuals) and female (69 individuals) participants had their photograph taken with a digital camera under variable outside lighting conditions. Participants were asked to pose with a neutral expression and to look directly into the camera. Head tilt and variation was evident for Hadza images and so images were selected by ACL on the basis of having a young adult appearance, a neutral expression, and they were looking directly the camera. Images were taken by FWM and the full set represented the majority of Hadza. Perceived age was used to select Hadza images and examining the composite images below show the average perceived ages. Verbal consent was obtained for all participants and the collection of photographs was approved by Harvard's ethics internal review board. Written consent was not obtained due to constraints in the field and posing for the photographs implies implicit consent.

### Measurements

**Figure 1:**
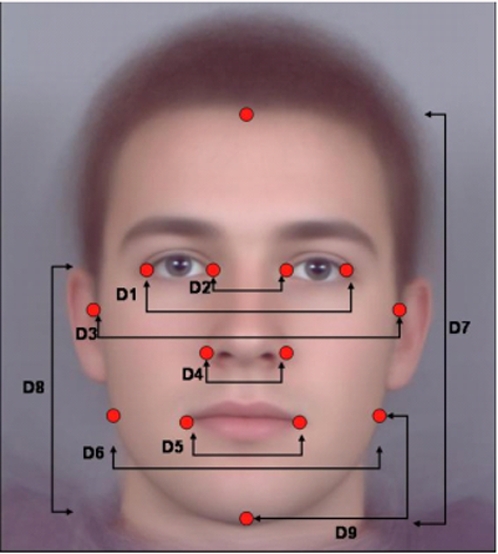
Measurements for symmetry and sexual dimorphism. Symmetry was calculated by taking left and right deviation from the midline, calculated from inter-pupillary distance, for points D1-D6 and then summing the absolute value of individual scores. Sexual dimorphism was measured by measuring distance between specific points and calculating four ratios based on these distances: Cheekbone Prominence (ChP, D3/D6), Jaw Height/Lower Face Height (JH/LFH, D9/D8), Lower Face Height/Face Height (LFH/FH, D8/D7), and Face Width/Lower Face Height (FW/LFH, D3/D8). All images were normalised on inter-pupillary distance.

We estimated horizontal asymmetry from x-y co-ordinates of 6 bilateral points following techniques used in previous studies [23], [24], [37] (see Figure 1). Briefly, symmetry was calculated by taking left and right deviation from the midline, calculated from inter-pupillary distance, for points and then summing the absolute value of individual scores. These symmetry measurements have been found to correlate with perceived measures of symmetry [24]. While pictures were initially screened for head tilt there was still the potential for outliers in facial asymmetry. For the full set, including all image types, mean asymmetry ranged from 5.8 to 187.7 with a mean of 50.0 and a standard deviation of 29.4. This suggested extreme values beyond two standard deviations (109) and so we adopted a conservative criterion of 120 to remove potential outliers. Any images with asymmetry scores higher than 120 were then excluded from the analysis for all sets. This removed 27 images from the original set of 874.

Sexual dimorphism measures were also taken from points marked on facial features (Figure 1). The identification of these features has been found to be reliable in previous studies [23], [37]. Following earlier studies, faces were standardised on interpupillary distance to eliminate variation in head distance from the camera. This is of particular importance for the Hadza and macaque images taken under non-standard conditions at varying camera distances. Colour differences between the images are irrelevant for measurements as they involve only shape information.

In total, four sexual dimorphism measurements were taken. These were Cheekbone Prominence (ChP, D3/D6), Jaw Height/Lower Face Height (JH/LFH, D9/D8), Lower Face Height/Face Height (LFH/FH, D7/D8), and Face Width/Lower Face Height (FW/LFH, D3/D8). These were found to be sexually dimorphic in the European sample here (see below) and in previous studies [24]. JH/LFH is a new measure here.

### Descriptives and distributions of scores

Descriptives for each variable split by image type and sex of image can be seen in Table S1. Kolmogorov-Smirnoff tests were used to test for normality of distribution (presented in Table S1). Significant deviation from normality was seen notably for asymmetry in the European sample in both men and women. This was the result of a skew towards low asymmetry for these measurements from these image sets.

### Fluctuating asymmetry and directional asymmetry

The six measures of asymmetry (D1 to D6) may display fluctuating asymmetry, (FA, right minus left approx 0) or directional asymmetry (DA, right minus left deviates from 0). We randomly selected 50 images from each grouping (male/female×macaque/ European/Hadza) so that each image set was equally represented in the following calculations. We calculated scores for right-left for each trait and conducted 1-sample t-tests against 0 to test for deviations. This revealed directional asymmetry for 4 traits. If traits exhibit DA then some individual variation may be due to heritable variation rather than being a measure of developmental stability [42]. We must then exercise some caution in concluding that such measures reflect only developmental stability. While the differences are significant, we do note that the proportions do not indicate uniformity of direction (i.e., it is not true that, for example, the distance from the inner eye to the midline is always greater on the right hand side of the face) . We note also the large sample sizes here allow us to see small effects and that there is a positive correlation between a composite score of FA and a composite score of DA traits (r = .174, *p* = .003) indicating the measures tap the same underlying factor. Most importantly, while 4 of the 6 traits demonstrate DA this does not mean that a significant proportion of the measure is DA. Our measure represents FA+DA. For each face we computed a second measure taking the difference from the average difference from the mean for each trait. For this score the mean is exactly 0 and represents an estimation of FA only, controlling for average genetic or other effects that cause the trait to be directional in nature. The correlation between our original measure and this second number for our sample is very high (r = .96, *p*<.001, r^2^ = .92) indicating that DA likely accounts for only 8% while FA accounts for 92% of the variance in our original measures. This suggests our measure largely reflects FA and not DA. See Table S2 for descriptive statistics of asymmetry.

### Sexual dimorphism in measures

Multivariate ANOVA's were carried out with sex of face as the fixed factor and masculinity measures as the dependent variables. For Europeans this revealed significant sexual dimorphism for all traits, with females scoring higher for FW/LFH (F_1,493_, = 57.2, *p*<.001) and ChP (F_1,493_, = 82.8, *p*<.001) and males scoring higher for JH/LFH (F_1,493_, = 53.0, *p*<.001) and LFH/FH (F_1,493_, = 45.6, *p*<.001). For Hadza this revealed significant sexual dimorphism for FW/LFH (F_1,134_, = 26.7, *p*<.001) and ChP (F_1,134_, = 8.1, *p* = .005), with females scoring higher for both these traits but no signifficant differences for JH/LFH (F_1,134_, = 0.1, *p* = .75) and LFH/FH (F_1,134_, = 0.4, *p* = .53). For macaques this revealed significant or near significant sexual dimorphism for all traits, with females scoring higher for ChP (F_1,214_, = 4.7, *p* = .031) and males scoring higher for JH/LFH (F_1,214_, = 9.3, *p* = .003), LFH/FH (F_1,214_, = 141.5, *p*<.001) and FW/LFH (F_1,214_, = 3.5, *p* = .061).

### Correlations between measures of masculinity and with symmetry

Tables S3, S4, and S5 show the correlations between all of the variables for each image set and for male and female images. The correlations with asymmetry are equivalent to the results of the regression analysis as only a single variable persists in each analysis.

### Making composite images

The 15 highest and lowest asymmetry scores for males and females were selected to make up the composites. For each set of 15 face images a single composite face was produced. The composite faces were created using specially designed software. Key locations (174 points) were manually marked around the main features and the outline of each face. The average location of each point in the 15 faces for each composite was then calculated. The features of the individual faces were then morphed to the relevant average shape before superimposing the images to produce a photographic quality result. For more information on this technique see [43], [44]. Composite images can be seen in Figure 1.

As the Hadza and the macaque images differed in lighting conditions we blended the shape and colour of the symmetric and asymmetric version together for each pair and then applied only the resultant colour to each original pair. This meant all images were standardised within pairs, so that both images possessed the same basic colouration. Images were also cropped to display only facial information.

An additional set of composite pairs were created for control purposes. These were made using the same methods as above but consisted of 15 randomly selected images from the appropriate groups. While random these images were labelled in the same manner (symmetric/asymmetric).

### Rating the composite images

#### Participants

50 individuals (27 female, mean age 28.8, SD = 6.7) judged the symmetric/asymmetric composites. 37 individuals judged the random composites (23 female, mean age 28.3, SD = 10.7). All individuals were volunteers responding to link on an electronic poster system and were UK based university students.

#### Procedure

Participants were administered a short questionnaire assessing age and sex before completing the face tests. The 6 pairs of symmetric and asymmetric faces of each sex were presented in separate blocks. Male faces were rated first, followed by female faces. Faces appeared on the screen side by side. Both order and side of presentation were randomised. Participants were asked to choose the face of the pair that they found most typical for that sex (i.e., for male faces: “which face appears most typical of males”). This action initiated the next face trial. A second set of participants completed the same trials but using the random composites.

## Results

### Measurements: composite measures of sexual dimorphism

In order for comparison amongst face type scores were standardised separately by face-type so that the mean for each group was 0 with a standard deviation of 1. An overall asymmetry score (sum of the absolute vales of deviation from midline for D1-D6) and an overall masculinity score ([JH/LFH+LFH/FH]-[ChP+ FW/LFH]) were calculated.

A univariate ANCOVA was conducted with asymmetry as the dependent variable, face-type (European/Hadza/Macaque) as a factor, and average masculinity as covariate. For female faces this revealed masculinity was not significantly related to asymmetry (F_1,452_ = 2.10, *p* = .148). Other effects and interactions were not significant (F_2,452_<2.44, *p*>.088). For male faces this revealed masculinity was significantly related to asymmetry (F_1,343_ = 12.09, *p*<.001). Other effects and interactions were not significant (F_2,343_<1.23, *p*>.295). Pearson product moment correlations between asymmetry and masculinity revealed that there was no significant correlation for female faces (r = −0.48, *p* = .285) and a significant negative correlation for males faces (r = −203, p<.001).

**Figure 2:**
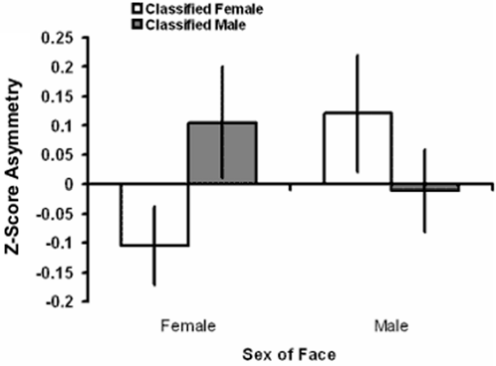
Asymmetry (+/− 1SE of mean) of faces classified as male or female in the discriminant analysis by sex of face. A significant interaction was found between sex of face and classification (F_1,835_ = 4.07 , *p* = .044) indicating that those correctly classified to their own sex were more symmetric than those misclassified to the opposite-sex.

As a secondary analysis we conducted a discriminant analysis using the four sexually dimorphic measures to discriminate sex of face separately for each face-type. Groups differed based on classification: European (Wilks' Lambda = .74, X^2^ = 148.98, DF = 4, *p*<.001), Hadza (Wilks' Lambda = .78, X^2^ = 33.11, DF = 4, *p*<.001), and macaque (Wilks' Lambda = .96, X^2^ = 8.25, DF = 4, *p* = .083). Classification was correct/incorrect: female 346/152, male 238/111. A univariate ANOVA was conducted with asymmetry as the dependent variable, and face-type (European/Hadza/Macaque), sex (male/female), and classification (male/female) as factors. This revealed a significant interaction between sex and classification (F_1,835_ = 4.07, *p* = .044). The interaction reflected that faces that were misclassified according to facial measures demonstrated greater asymmetry than faces that were classified as sex typical (see Figure 2). A theoretically unrelated significant interaction between face-type and classification was also found (F_1,835_ = 4.37, *p* = .012). Other effects and interactions were not significant (F_1/2,343_<1.22, *p*>.296).

### Measurements: regression of sexually dimorphic traits by sex and face-type

Overall asymmetry score was predicted using the four individual measures of sexual dimorphism (see Methods) entered into a backwards linear regression analysis (*p* = .1 criteria, only the final model is reported here). Measures of sexual dimorphism were treated separately as correlations between these traits were generally low. For full interrelationships between measures of symmetry and sexual dimorphism see Tables S3, S4, and S5.

For European faces, the model was close to significant for females (F_1,316 = _3.1, *p* = .080, R^2^ = .01) where the masculine trait LFH/FH was positively related to asymmetry (β = .10, *p* = .080). The model for males was significant (F_1,175 = _6.6, *p* = .011, R^2^ = .04) where the masculine trait JH/LFH was negatively related to asymmetry (β = −.19, *p* = .011).

For Hadza faces, the model was not significant for females with no significant predictors (all *p*>.23) but was significant for males (F_1,65 = _7.1, *p* = .010, R^2^ = .10), where the masculine trait JH/LFH was negatively related to asymmetry (β = −.31, *p* = .010).

For macaque faces, the model revealed a significant model for females (F_1,109 = _4.6, *p* = .035, R^2^ = .04), where the masculine trait JH/LFH was positively related to asymmetry (β = .20, *p* = .035). The model for males was also significant (F_1,103 = _4.0, *p* = .047, R^2^ = .04), where the masculine trait LFH/FH was negatively related to asymmetry (β = −.19, *p* = .047).

The results of this analysis are robust to corrections for multiple tests (see Text S1, Table S6).

### Perception of composites

**Figure 3:**
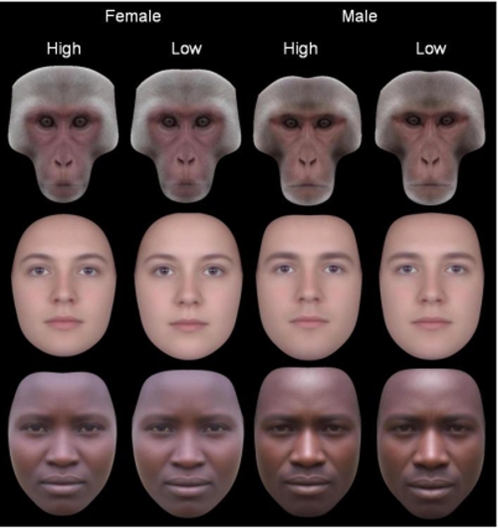
High and low symmetry composite faces for macaques, Hadza, and Europeans. All images are normalised on inter-pupillary distance to control relative image size, have been made perfectly symmetric, and each high/low pair possesses the average colour information of both. Perceptual differences are then dependent on shape differences between high and low symmetry faces that are independent of symmetry.

**Figure 4:**
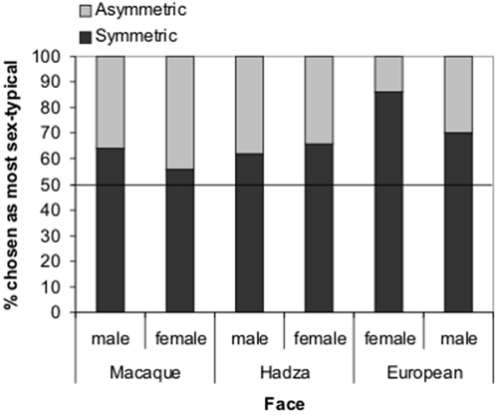
Proportion of individuals choosing high and low symmetry composite faces for macaques, Hadza, and Europeans as most sex-typical (i.e. masculine for males, feminine for females).

Measured sexual dimorphism may not capture all aspects of this trait to which humans are visually sensitive. To examine perception, composite images of individuals with high and low facial asymmetry were created for males and females of each population (see Methods, Figure 3). These image pairs were shown to European human participants, who were asked out of the pair which was more typical of their sex in appearance. Chi square tests were conducted on the proportions showing that, for females, symmetric Hadza (χ^2^ = 5.1, *p* = .021) and Europeans (χ^2^ = 25.9, *p*<.001) were selected as more typically female than asymmetric Hadza and Europeans. Proportions were not significantly different for female symmetric and asymmetric macaques (χ^2^ = 0.7, *p* = .40). For males, symmetric Hadza (χ^2^ = 2.9, *p* = .088, *p* = .044 one-tailed as predicted from measurement data), macaques (χ^2^ = 3.9, *p* = .048), and Europeans (χ^2^ = 8.0, *p* = .005) were selected as more typically male than asymmetric Hadza, macaques, and Europeans. Proportions can be seen in Figure 4. A binomial test revealed that the proportion of symmetric images being chosen as most sexually dimorphic significantly differed from chance (chosen = 6/6, chance 3/6, *p* = .031).

Comparing the overall scores to chance (50%) using one-sample t-tests revealed that the choice of symmetric/asymmetric composites differed from chance (mean = 67%, SD = 17%, t_49_ = 7.01, *p*<.001) while the random composites did not (mean = 47%, SD = 17%, t_36_ = 7.01, *p* = .337). An independent-samples t-test revealed a significant difference in choice between symmetric/asymmetric and random composites (t_85_ = 5.36, *p*<.001). Thus the overall pattern for the composites was that symmetric images were seen as more sexually dimorphic in humans and male macaques using both chance and a control set of images as criterion.

## Discussion

Our results indicate that symmetry and sexually dimorphic traits are related in male and female faces in humans, in a modern western society and in a different society living under conditions better approximating human evolutionary history, and across species, both in humans and a non-human primate. We found symmetry was related to sexual dimorphism using physical measurements of large numbers of faces and perceptual tests based on the perceived sexual dimorphism of faces that were most and least symmetric in our samples. We note that only European participants provided the ratings of the composites and it is likely difficult for them with limited experience to judge masculinity in Hadza and macaque faces. In fact this raises an interesting point. The generally consistent judgement that symmetric individuals appeared more sexually dimorphic across all face types from European judges that there is some commonality in features that cross culture and species.

We note that the measurements may not necessarily capture sexual dimorphism fully (as suggested by the discriminant analysis) but that together the patterns of the measurement and perceptual data supports the notion that sexual dimorphism and symmetry in faces are linked. We also note that some caution must be taken in interpretation as our symmetry measurements do not all fully fulfil the criteria for fluctuating asymmetry, though appear to mainly capture FA and not DA (see Methods). The DA in our measures might reflect expressive habits, for example, natural smiles are asymmetric reflecting hemispheric specialisation in the control of emotion [45]. We also note that the different types of analysis reveal some differences in sex effects as sexual dimorphism was not found to be related to symmetry using an additive measure whereas a relationship emerged in the discriminant analysis. The overall pattern, however, is that symmetry was related to some aspect of dimorphism either via one aspect of measurement: overall additive or discriminative measurements, individual trait measures, or perceptual measures.

If sexual dimorphism and symmetry in faces advertise quality in both males and females then only high quality males can grow symmetric and masculine and high quality females can grow symmetric and feminine. Similar arguments have been put forward to explain co-variation between trait size and symmetry in birds [13]. This relationship then suggests that notions of symmetry and sexual dimorphism signalling a single aspect of quality are true. We also note, however, that the relationship is not absolute, leaving the potential that both may also signal other separable qualities. Symmetry and sexual dimorphism may then be seen as complementary signals of the same quality, but may also signal other qualities independently. Previous studies have shown negative associations between symmetry and trait size in the secondary sexual traits of a variety of taxa, including birds and primates [3], [13]. The results here demonstrate that faces are involved in selection with no obvious association with weaponry involved in intra-sexual selection, as shown in previous studies of primate tooth dimorphism. Bare skin on faces in primate species is common [46], further highlighting the potential role for sexual selection acting on faces across the primate lineage.

Sexual dimorphism is facilitated by sex hormones [47]. Symmetry is linked to developmental stability [16]. Symmetry and sexual dimorphism may be linked by an underlying biological factor. For example, both may reflect gene quality. If high quality genes are those that code, potentially, for efficient immune systems, high metabolic efficiency, or even behavioural traits that secure resources for an organism during development, then such genes may also allow an organism to grow both symmetric and sexually dimorphic. By measuring how well an organism can cope with genomic stress and environmental perturbations, symmetry may be an honest signal of gene-quality given that studies show that such stressors during development increase asymmetry [48]. The link between sexual dimorphism and good-genes advertisement has produced many more theories. Honest signalling in this case might arise through an immuno-competence handicap mechanism [49], whereby sex hormones represent a behavioural or immunological handicap to the organism. Other mechanisms may also create honesty in hormone mediated traits, for example via cortisol levels [50]. Theoretically, honesty can also arise, when high-quality individuals achieve greater benefit from an allocation to a trait than do low-quality individuals even when the costs of the trait are equivalent [51]. Mate choice based on symmetry and sexual dimorphism may then provide indirect benefits, acquiring good-genes from partners that benefit offspring, or direct benefits, acquiring factors other than good-genes from partners that benefit the choosing individual, such as resources. Of course there are other potential benefits of sexual dimorphism and symmetry, for example fertility [19], [31]. Ultimately it may be unnecessary to consider the relative weights of indirect and direct benefits as they are difficult to tease apart. For example, males with good-genes for immunity may also be most able to provide food or defend a large, high quality territory; thus selection for good resources/behaviour may reflect selection for good-genes.

The current study shows that symmetry and sexual dimorphism are related in both male and female faces across cultures and species. Examining the regression models suggests that the relationship between symmetry and sexual dimorphism is stronger for males than for females for both the European and Hadza samples; Hadza males also retain symmetry with age more than females do [52]. In the additive measures, symmetry was related to dimorphism only for males, but the discriminant measure was related in females. Our perceptual test may be biased in examining sex differences as it is dependent on the number of images in the sample. For example, we may see the largest effect in females in the European sample potentially because we had the largest number of participants in this group, making the composites more likely to represent the extremes of asymmetry. Following the regression models then, we do see a more consistent effect in male faces. The immuno-competence-handicap hypothesis was originally proposed for males and there is reasonable evidence testosterone reduces immune function [32]. Weaker relationships for symmetry and femininity in females may stem from the fact that the relationship between oestrogen and immuno-competence appears weaker than between testosterone and immuno-competence. In humans, higher oestrogen is linked to development of cancers [53], suggestive of a reduction in immune function, although animal studies suggest that while suppressing cell-mediated immunity, oestrogen may enhance humoral immunity [54]. As feminine facial traits differ less from immature traits than do male traits [28], they are also potentially less costly to produce. Taken together these findings suggest that feminine traits may be less powerful signals of good-genes than masculine traits, although we note there that here femininity in female faces is correlated with symmetry, another proposed aspect of quality. Additionally, our data does not necessarily support the idea that sexual dimorphism represents a single continuum in faces. We generally found relatively weak correlations amongst dimorphism measures (see Tables S3, S4, and S5). Here perhaps we have evidence that certain face traits may be more involved in sexual selection than others.

While studies demonstrate that preferences can arise via experience [55], [56], as a by-product of pattern recognition in the visual system works without either trait being related to quality, such reasoning does not predict co-variation between traits in natural populations. It has also been suggested the preference for symmetry of tails in bird species may in fact be due to aerodynamics and not developmental stress [17]. While this would be plausible for a species in which small deviations in symmetry may have large effects, as is the case for flying, it is difficult to imagine such small deviations in symmetry would impact on motor action in faces so much as to appear unattractive. Such views imply that symmetry and sexual dimorphism preferences are arbitrary and neither view proposes underlying mechanisms that would influence the development of both.

In conclusion, our finding of sex specific co-variation with symmetry, femininity for females, masculinity for males, indicates then that both sexual dimorphism and symmetry likely are signals advertising quality. We have shown such a relationship in diverse human cultures and in a monkey species, which suggests that signalling properties of faces are universal across human populations and that facial advertisements of quality may have arisen relatively early in the phylogeny of primates.

## Supporting Information

Table S1 Descriptive statistics for measured traits
(0.07 MB DOC)

Table S2 Tests for directional asymmetry for the 6 symmetry traits
(0.03 MB DOC)

Text S1 Iterated bonferonni correction of p-values for regression analysis
(0.03 MB DOC)

Table S3 Correlations amongst measures of sexual dimorphism and Symmetry for macaque sample (female/male).
(0.03 MB DOC)

Table S4 Correlations amongst measures of sexual dimorphism and Symmetry for European sample (female/male).
(0.03 MB DOC)

Table S5 Correlations amongst measures of sexual dimorphism and Symmetry for Hadza sample (female/male).
(0.03 MB DOC)

Table S6 Comparison of directional p-values with iterated Bonferonni corrected significance levels.
(0.03 MB DOC)
