# Supplementary material for: Symmetry Is Related to Sexual Dimorphism in Faces: Data Across Culture and Species
Source: PLoS One. 2008 May 7;3(5):e2106. doi: 10.1371/journal.pone.0002106 (PMC2329856; doi:10.1371/journal.pone.0002106)
Supplement: Table S1 — Descriptive statistics for measured traits (0.07 MB DOC) [file pone.0002106.s001.doc]

**Table S**1: Descriptive statistics for measured traits

| Image type | sex |  | N | Minimum | Maximum | Mean | Std. Deviation | Kolmogorov-Smirnov |
| --- | --- | --- | --- | --- | --- | --- | --- | --- |
| Macaque | female | Asym | 111 | 13.31 | 119.51 | 62.08 | 25.42 | NS |
|  |  | ChP | 111 | 0.87 | 1.14 | 1.01 | 0.06 | NS |
|  |  | LFH/FH | 111 | 0.54 | 0.67 | 0.60 | 0.02 | NS |
|  |  | JH/LFH | 111 | 0.14 | 0.31 | 0.22 | 0.03 | NS |
|  |  | FW/LFH | 111 | 1.06 | 1.45 | 1.28 | 0.08 | NS |
|  | male | Asym | 105 | 15.71 | 111.12 | 67.97 | 24.78 | NS |
|  |  | ChP | 105 | 0.90 | 1.26 | 1.02 | 0.06 | NS |
|  |  | LFH/FH | 105 | 0.57 | 0.69 | 0.64 | 0.02 | NS |
|  |  | JH/LFH | 105 | 0.15 | 0.32 | 0.23 | 0.03 | NS |
|  |  | FW/LFH | 105 | 1.04 | 1.50 | 1.26 | 0.09 | NS |
| European | female | Asym | 318 | 5.79 | 112.20 | 39.01 | 18.86 | 0.02 |
|  |  | ChP | 318 | 1.06 | 1.26 | 1.15 | 0.04 | NS |
|  |  | LFH/FH | 318 | 0.54 | 0.91 | 0.61 | 0.04 | <.001 |
|  |  | JH/LFH | 318 | 0.31 | 0.48 | 0.40 | 0.02 | NS |
|  |  | FW/LFH | 318 | 1.01 | 1.37 | 1.19 | 0.07 | NS |
|  | male | Asym | 177 | 10.89 | 113.29 | 39.35 | 19.73 | 0.03 |
|  |  | ChP | 177 | 1.03 | 1.25 | 1.12 | 0.04 | NS |
|  |  | LFH/FH | 177 | 0.57 | 0.71 | 0.63 | 0.03 | NS |
|  |  | JH/LFH | 177 | 0.34 | 0.53 | 0.42 | 0.03 | NS |
|  |  | FW/LFH | 177 | 0.93 | 1.33 | 1.14 | 0.07 | NS |
| Hadza | female | Asym | 69 | 9.47 | 117.24 | 48.89 | 27.22 | NS |
|  |  | ChP | 69 | 1.02 | 1.18 | 1.11 | 0.04 | NS |
|  |  | LFH/FH | 69 | 0.55 | 0.68 | 0.61 | 0.02 | NS |
|  |  | JH/LFH | 69 | 0.31 | 0.45 | 0.36 | 0.03 | NS |
|  |  | FW/LFH | 69 | 1.10 | 1.36 | 1.24 | 0.06 | NS |
|  | male | Asym | 67 | 15.00 | 103.08 | 45.89 | 21.08 | NS |
|  |  | ChP | 67 | 1.02 | 1.17 | 1.09 | 0.03 | NS |
|  |  | LFH/FH | 67 | 0.55 | 0.67 | 0.60 | 0.03 | NS |
|  |  | JH/LFH | 67 | 0.24 | 0.43 | 0.36 | 0.04 | NS |
|  |  | FW/LFH | 67 | 1.05 | 1.34 | 1.18 | 0.07 | NS |
