# Supplementary material for: Symmetry Is Related to Sexual Dimorphism in Faces: Data Across Culture and Species
Source: PLoS One. 2008 May 7;3(5):e2106. doi: 10.1371/journal.pone.0002106 (PMC2329856; doi:10.1371/journal.pone.0002106)
Supplement: Table S2 — Tests for directional asymmetry for the 6 symmetry traits (0.03 MB DOC) [file pone.0002106.s002.doc]

**Table S2: Tests for directional asymmetry for the 6 symmetry traits**

|  | N | Mean | Std. Deviation | Kolmogorov-Smirnov | Proportion of cases right > left | t |
| --- | --- | --- | --- | --- | --- | --- |
| D1 | 300 | 0.8 | 2.3 | NS | 62% | 5.8** |
| D2 | 300 | 0.6 | 2.9 | NS | 59% | 3.3** |
| D3 | 300 | -4.1 | 9.1 | NS | 32% | -7.8** |
| D4 | 300 | 0.4 | 6.4 | NS | 51% | 1.2 |
| D5 | 300 | 1.2 | 7.0 | NS | 42% | 2.8* |
| D6 | 300 | 0.1 | 8.9 | NS | 50% | 0.2 |

*p<.05, **p<.001
