# Supplementary material for: Symmetry Is Related to Sexual Dimorphism in Faces: Data Across Culture and Species
Source: PLoS One. 2008 May 7;3(5):e2106. doi: 10.1371/journal.pone.0002106 (PMC2329856; doi:10.1371/journal.pone.0002106)
Supplement: Text S1 — Iterated bonferonni correction of p-values for regression analysis (0.03 MB DOC) [file pone.0002106.s003.doc]

*Text S1: Iterated bonferonni correction of p-values for regression analysis*

It is noted that conducting 24 correlations may result in spurious acceptance of significance. While we report the results of a regression, our analysis is equivalent to running correlations between asymmetry and the masculine measures for each image group. Here we compare p-values of the five significant or near significant results from the regression analyses (which are equivalent to the p-values of the correlations as only one trait correlated with asymmetry in each case) to corrected significance levels. Table S6 shows 1-tailed p values compared with sequential Bonferonni corrected levels of significance (Rice, 1989). This is done by ordering p-values by decreasing levels of significance and comparing with a Bonferroni corrected p based the number of tests conducted (i.e., p1 compared to 0.05, p2 compared to 0.05/2, p3 compared to 0.05/3). 1-tailed values are used here as all significant correlations were in the predicted directions. As can be seen in Table S6, only one value does not survive correction and misses the new criteria by only 0.001. We note that strictly we should stop at the first non-significant value but here the difference between the p value and corrected significance level is marginal and so we present all tests.

**Reference**

Rice, W. R. 1989 Analyzing tables of statistical tests. *Evolution* 43, 223-225.
