# Supplementary material for: Symmetry Is Related to Sexual Dimorphism in Faces: Data Across Culture and Species
Source: PLoS One. 2008 May 7;3(5):e2106. doi: 10.1371/journal.pone.0002106 (PMC2329856; doi:10.1371/journal.pone.0002106)
Supplement: Table S3 — Correlations amongst measures of sexual dimorphism and Symmetry for macaque sample (female/male). (0.03 MB DOC) [file pone.0002106.s004.doc]

**Table S3: correlations amongst measures of sexual dimorphism and**

**Symmetry for macaque sample (female/male).**

*p<.05, **p<.001

| **Trait** | LFH/FH | JH/LFH | FW/LFH | Asymmetry |
| --- | --- | --- | --- | --- |
| ChP | -0.13/0.15 | 0.05/0.17 | 0.50**/0.63** | 0.00/-0.12 |
| LFH/FH | - | 0.45**/0.46** | -0.36/-0.10 | 0.05/-0.19* |
| JH/LFH | - | - | 0.07/0.30** | 0.20*/-0.12 |
| FW/LFH | - | - | - | 0.06/-0.07 |
