# Supplementary material for: Symmetry Is Related to Sexual Dimorphism in Faces: Data Across Culture and Species
Source: PLoS One. 2008 May 7;3(5):e2106. doi: 10.1371/journal.pone.0002106 (PMC2329856; doi:10.1371/journal.pone.0002106)
Supplement: Table S4 — Correlations amongst measures of sexual dimorphism and Symmetry for European sample (female/male). (0.03 MB DOC) [file pone.0002106.s005.doc]

**Table S4: correlations amongst measures of sexual dimorphism and**

**Symmetry for European sample (female/male).**

| **Trait** | LFH/FH | JH/LFH | FW/LFH | Asymmetry |
| --- | --- | --- | --- | --- |
| ChP | -0.06/-0.03 | -0.13*/-0.20* | 0.13*/0.00 | 0.00/0.02 |
| LFH/FH | - | 0.10τ/0.17* | -0.37**/-0.32** | 0.02/-0.11 |
| JH/LFH | - | - | -0.05/-0.05 | 0.10τ/-0.19* |
| FW/LFH | - | - | - | 0.04/-0.03 |

τ< p<.10, *p<.05, **p<.001
