# Supplementary material for: Symmetry Is Related to Sexual Dimorphism in Faces: Data Across Culture and Species
Source: PLoS One. 2008 May 7;3(5):e2106. doi: 10.1371/journal.pone.0002106 (PMC2329856; doi:10.1371/journal.pone.0002106)
Supplement: Table S5 — Correlations amongst measures of sexual dimorphism and Symmetry for Hadza sample (female/male). (0.03 MB DOC) [file pone.0002106.s006.doc]

**Table S5: correlations amongst measures of sexual dimorphism and**

**Symmetry for Hadza sample (female/male).**

*p<.05, **p<.001

| **Trait** | LFH/FH | JH/LFH | FW/LFH | Asymmetry |
| --- | --- | --- | --- | --- |
| ChP | 0.24*/0.12 | -0.07/0.11 | 0. 02/0.18 | 0.10/0.01 |
| LFH/FH | - | 0.00/0.27* | -0.29*/-0.41** | -0.15/0.04 |
| JH/LFH | - | - | -0.26*/-0.17 | 0. 07/-0.31* |
| FW/LFH | - | - | - | -0.04/0.06 |
