# Supplementary material for: Symmetry Is Related to Sexual Dimorphism in Faces: Data Across Culture and Species
Source: PLoS One. 2008 May 7;3(5):e2106. doi: 10.1371/journal.pone.0002106 (PMC2329856; doi:10.1371/journal.pone.0002106)
Supplement: Table S6 — Comparison of directional p-values with iterated Bonferonni corrected significance levels. (0.03 MB DOC) [file pone.0002106.s007.doc]

**Table S**6: comparison of directional p-values with iterated Bonferonni corrected significance levels.

|  | test | Original p | 1-tailed p | Corrected significance |
| --- | --- | --- | --- | --- |
| 1 | European female | 0.080 | 0.040* | 0.050 |
| 2 | Macaque male | 0.047 | 0.024* | 0.025 |
| 3 | Macaque female | 0.035 | 0.018 | 0.017 |
| 4 | European male | 0.011 | 0.006* | 0.013 |
| 5 | Hadza Male | 0.010 | 0.005* | 0.010 |

*lower than corrected significance
